# Correction: Revealing the photocatalytic dissociation of water molecules on rutile TiO_2_ surface *via* hybrid functional based linear response time-dependent density functional theory

**DOI:** 10.1039/d5sc90202a

**Published:** 2025-09-19

**Authors:** Lei Wang, Xiaofeng Liu, Qunxiang Li, Jinlong Yang, Wei Hu

**Affiliations:** a Department of Chemical Physics, State Key Laboratory of Precision and Intelligent Chemistry, University of Science and Technology of China Hefei Anhui 230026 China liqun@ustc.edu.cn whuustc@ustc.edu.cn; b School of Physics, Hefei University of Technology Hefei Anhui 230009 China lxf@hfut.edu.cn

## Abstract

Correction for ‘Revealing the photocatalytic dissociation of water molecules on rutile TiO_2_ surface *via* hybrid functional based linear response time-dependent density functional theory’ by Lei Wang *et al.*, *Chem. Sci.*, 2025, https://doi.org/10.1039/d5sc02736e.

The authors regret that an email address was missing for corresponding author Wei Hu. The email address for correspondence is whuustc@ustc.edu.cn.

The Royal Society of Chemistry apologises for these errors and any consequent inconvenience to authors and readers.

